# Mapping and Validation of Peptides Differentially Recognized by Antibodies from the Serum of *Yellow Fever Virus*-Infected or 17DD-Vaccinated Patients

**DOI:** 10.3390/v14081645

**Published:** 2022-07-27

**Authors:** Eneida Santos Oliveira, Naiara Clemente Tavares, Stella Garcia Colombarolli, Izabella Cristina Andrade Batista, Camila Sales Nascimento, Philip Louis Felgner, Rafael Ramiro de Assis, Carlos Eduardo Calzavara-Silva

**Affiliations:** 1 Gerência da Rede Ambulatorial Especializada, Secretaria Municipal de Saúde de Belo Horizonte, Prefeitura de Belo Horizonte, 2336 Afonso Pena Avenue, Belo Horizonte 301300-007, Brazil; eneida.oliveira@pbh.gov.br; 2 Grupo de Imunologia Celular e Molecular, Instituto Rene Rachou, Fiocruz, 1715 Augusto de Lima Avenue, Belo Horizonte 30190-002, Brazil; naiara.paula@fiocruz.br (N.C.T.); stellacolombarolli29@gmail.com (S.G.C.); izabella.batista@fiocruz.br (I.C.A.B.); cnascimento@aluno.fiocruz.br (C.S.N.); 3 Istituto di Scienze e Tecnologie Chimiche “Giulio Natta”, National Research Council of Italy, 12 Alfonso Corti, 20133 Milano, Italy; 4 Vaccine Research and Development Center, Department of Physiology, University of California, Irvine 3052 Hewitt Hall, Irvine, CA 92697, USA; pfelgner@hs.uci.edu (P.L.F.); rramirod@hs.uci.edu (R.R.d.A.)

**Keywords:** peptide microarray, flavivirus, differential diagnosis, *Yellow Fever virus*

## Abstract

Yellow Fever disease is caused by the *Yellow Fever virus* (YFV), an arbovirus from the *Flaviviridae* family. The re-emergence of Yellow Fever (YF) was facilitated by the increasing urbanization of sylvatic areas, the wide distribution of the mosquito vector, and the low percentage of people immunized in the Americas, which caused severe outbreaks in recent years, with a high mortality rate. Therefore, serological approaches capable of discerning antibodies generated from the wild-type (YFV-WT) strain between the vaccinal strain (YFV-17DD) could facilitate vaccine coverage surveillance, enabling the development of strategies to avoid new outbreaks. In this study, peptides were designed and subjected to microarray procedures with sera collected from individuals infected by WT-YFV and 17DD–YFV of YFV during the Brazilian outbreak of YFV in 2017/2018. From 222 screened peptides, around ten could potentially integrate serological approaches aiming to differentiate vaccinated individuals from naturally infected individuals. Among those peptides, one was synthesized and validated through ELISA.

## 1. Introduction

The *Yellow Fever virus* (YFV) is a virus from the *Flavivirus* genus and is characterized by an acute, febrile, hemorrhagic infectious disease, causing severe liver damage [1]. YFV is an enveloped virus with a positive-sense single-stranded RNA genome coding for a precursor polyprotein composed of three structural (capsid, C; precursor membrane and membrane, prM/M; envelope, E) and seven non-structural proteins (NS1, NS2A, NS2B, NS3, NS4A, NS4B, and NS5) [1,2]. The E protein, organized into three domains (EDI, EDII, and EDIII), is considered the main exposed antigen and crucial to binding and entry into target cells, triggering an effective immune response and inducing neutralizing antibody production [3,4]. The C protein is associated with the viral RNA and implicated in its condensation, and the prM/M protein protects the E protein from having conformational modifications due to the acid environment on secretory pathways [5,6]. Non-structural proteins are mostly related to genome replication and virion assembly. Besides its role in virus replication, the NS1 is secreted into circulation and considered a flaviviruses infection biomarker. In addition, reports show that NS1, NS2A, NS4B, and NS5 are involved in immune system modulation and evasion. [3,7,8,9,10]. Naturally, YFV is maintained in a sylvatic cycle, involving non-human primates as hosts and vectors of the genera *Haemagogus*, *Sabethes,* and *Aedes*. Two other types of YFV transmission cycles are also presented—the intermediate cycle—in which mosquitoes reproduce in nature and infect human and non-human primates in villages located in rural regions and urban areas. These are characterized by YFV transmissions mainly from *Aedes aegypti*, which breed in city areas, in densely populated areas, and mostly among people with little or no immunity. The signs of Yellow Fever (YF) disease begin three to six days after YFV transmission to the human host. The first symptoms appear to be very generic and common to other diseases, such as fever, muscle pain, headache, joint pain, and nausea. In the next 48 h, 15% of infected people develop the classic signs of YF caused by intoxication during the virus replication mainly in the liver. These signs are jaundice, bleeding, high fever, dark urine, shock, and organ failure. As the name implies, people infected with YFV become yellow due to liver dysfunction. There are no specific antiviral treatments and, therefore, treatment is mainly based on palliative care, which results in a significant mortality rate between 20% and 50% of individuals who develop a severe YF disease [1,11]. 

Since an attenuated vaccine against YFV was developed [12], and through extensive vaccination and vector control campaigns, the incidence of YF cases was significantly reduced. Even if a vaccine specific for YF is available, in recent years South America has undergone a re-emergence of YF, and Brazil exhibits recurring epidemics characterized by high numbers of cases. This may be associated with the sylvatic cycle, which has been altered with the increased involvement of humans in the progressive urbanization of wild areas, extensive spread of the mosquito vector, and low immunization coverage. This re-emergence of YF has led to a scenario characterized by several outbreaks, with high mortality rates in the Americas and Africa [1,13,14]. Further, the risk of re-urbanization or environmental disasters sustained by vector plasticity in some territories and large susceptible populations without vaccination routine explains the recurrent epidemics in endemic regions such as Africa and South America [15]. 

Currently, the YF vaccine used in Brazil is a live-attenuated 17DD virus vaccine (17DD-YFV), a substrain derived from the 17D virus [16]. YF occurs in Brazil due to the YFV maintenance in enzootic and sylvatic cycles [17]. Since 1933, Brazil has been experiencing sylvatic YF outbreaks, with cases reported throughout the country until 1964 [18,19]. Over the years, the sylvatic YF cases were centralized in north and midwest regions, and from 1999 the majority of them began to occur in the southeast, midwest, and south areas [17,20]. From December 2016 to 2018, the country experienced a YF outbreak with several cases in low-endemic areas such as the southeast region [21,22,23,24]. The most affected areas were near large urban centers densely populated with low vaccination coverage [24]. Additionally, these regions underwent an infestation of the vector mosquitoes and climate changes, developing a scenario that could promote the re-emergence of YF in urban areas [25]. In this regard, Brazilian health authorities extended the immunization coverage and issued recommendations to the entire country for YF vaccination, including children [17,26]. Yet, the recognition of individuals that present antibodies against the vaccinal strain and its differentiation from antibodies produced after natural infection of the wild-type (WT-YFV) are still a limitation.

The determination of specific antibodies that allow the differentiation among the WT-YFV and vaccinal strain would enable the development of serological approaches capable of diagnosing YFV natural infection, discriminating antibodies against WT-YFV and the vaccinal strain, as well as recognizing immunized individuals. These methods would support the health professionals in the correct management of infected individuals and the vaccinal coverage attestation, impacting the morbidity and mortality reduction, as the prevention of new YF outbreaks. Thus, intending to identify and validate peptides capable of being differentially recognized by antibodies of WT-YFV naturally infected patients, as well as 17DD-YFV vaccinated individuals, we designed antigenic peptides based on epitopes from the wild and vaccine strains of YFV to be screened by using the peptide microarray approach and validated through enzyme-linked immunosorbent assay.

## 2. Materials and Methods

### 2.1. Samples

Human serum samples were collected during the Brazilian outbreak of YFV in 2017/2018 in the Eduardo de Menezes Hospital, Minas Gerais, Brazil. Patient vaccination certificates, clinical records, and patients’ self-reports were used to define if an individual was previously vaccinated and/or recently infected by YFV. Recent YFV infections were confirmed through molecular (PCR) and serological (YFV-IgM) diagnostic tests, following the Hospital’s clinical routine. Samples collected from asymptomatic volunteers, negative in both tests, with no records of YFV-vaccination or recent infection were used to compose the “Negative-YFV group” (*n* = 30); symptomatic patients testing as “positive” at least in one diagnostic test and with no record of YFV-vaccination were considered YFV-infected and non-vaccinated and their samples were used to compose the “WT-YFV group” (*n* = 27); samples collected from volunteers testing as “negative” in both diagnosis test and with a record of YFV-vaccination were used to compose the “17DD-YFV group” (*n* = 30). Vaccinated YFV-reagent individuals before the onset of symptoms, during clinical manifestations, or after hospital discharge, were excluded from the present study.

### 2.2. Design of Peptides

Specific peptides to WT-YFV and 17DD-YFV were designed according to described by Bonaldo et al., 2017 and Gómez et al., 2018 [27,28] that observed high conservation in the sequences of circulating YFV in the 2016–2017 outbreak in the southeast region of Brazil. Accordingly, here we used the full coding sequence of the polyprotein of ten isolates from this outbreak available at GenBank (accession numbers AVQ67911.1, AVQ67912.1, AVQ67913.1, AVQ67914.1, AVQ67915.1, AVQ67916.1, AVQ94372.1, AVQ94369.1, AVQ94370.1, AVQ94371.1) [29] and the prototype sequence ES-505 [29]. The sequences were aligned using the ClustalW software (Version 1.81, Conway Institute UCD, Dublin, Ireland) [30], using the default parameters. An identity matrix was constructed using SIAS server (BLOSUM62 matrix with a penalty for creating a gap of 10 and a penalty for gap extension of 0.5) [31]. The identity values were calculated according to the formula:

$$identity\;\left(ID\right)\;\%=\frac{num.\;identical\;amino\;acid}{Sequence\;size}$$

In summary, at least when analyzed within the scope of their amino acid sequences, the circulation YFV have practically identical proteomes (greater than 99%). For this reason, the prototypical sequence of this outbreak (Genbank: KY885001.2, as described by Bonaldo et al., 2017 [27]), here named YFV-WT, was used as a model for the peptide design.

Similar to the strategy described by Mishra et al., 2018 [32], we proposed the construction of a peptide microarray that encompasses all the amino acid differences identified between the vaccine strain and the wild-type circulating strains of YFV. However, for this purpose, the strategy employed was to only use peptides that contain at least one amino acid that differentiates YFV-17DD from YFV-WT.

Initially, to identify the differences in serological response against vaccine antigens and antigens from circulating strains in the recent YF outbreak, the differences in the protein sequences among the YFV vaccine strains were evaluated. Firstly, a comparison was made between the sequences of the strain used for vaccine production in Brazil (YFV-17DD, Genbank: U17066.1) [29] and the 17D-204 strain (Genbank: MN708488.1) [29]. Due to the high identity between the sequences of YFV-17D and YFV-17DD, as well as the fact that the vaccine strain in Brazil is the YFV-17DD, only the YFV-17DD sequence was maintained as a reference sequence of the vaccine strain for this study.

Then, a comparison of YFV-17DD and YF-WT sequences was performed. Initially, the sequences were aligned using the ClustalW software (as described above), and differences in the amino acid sequence were identified. 

Peptide databases whose sequence could differentiate between YFV-17DD and YFV-WT viruses and peptide designs were performed according to Colombarolli et al., 2022 [33]. 

To explore the antigenic potential of the selected peptides, linear epitope prediction was performed using the polyprotein sequence of the YFV-WT strain. The prediction was performed using the Bepipred 2.0 algorithm (Version 2.0, Department of Health Technology, Lyngby, Denmark) [34] with a threshold value of 0.5. For comparison purposes, peptides in which the differential amino acid is located within an epitope region predicted by the algorithm were considered potentially more immunogenic. 

### 2.3. Microarray Procedures

The microarrays were printed by the PEPperPRINT company (PEPperPRINT, Rischerstraße, Germany) onto nitrocellulose pads on glass slides, to a total of16 pads per slide. In addition to the designed Yellow Fever peptides, all printed as triplicate spots, the arrays were designed to contain the following controls: (1) blank spots, on which only a glycine residue was imprinted, (2) reaction/labeling control spots, composed by a 10-mer peptide (YPYDVPDYAG) derived from the Influenza virus hemagglutinin protein (HA), thus, serving as positive anti-HA-Cy5 (clone 12CA5) antibody control spots, and (3) binding control spots, composed by a 14-mer (KEVPALTAVETGAT) from poliovirus as positive sera reactivity control. The slides were kept protected from light and with silica to protect from humidity up to the moment of use.

The microarray assays, using human sera, were performed using the samples and groups described in Section 2.1.

All microarray procedures were performed according to the methodology described by Colombarolli et al., 2022 [33].

### 2.4. Enzyme-Linked Immunosorbent Assay (ELISA)

To further validate the microarray findings, the peptide selected was then custom synthesized by the company Biomatik (Biomatik Corporation, Kitchener, ON, Canada) for ELISA assay. For this assay, a subset of the sera samples from the microarrays was used (Negative samples *n* = 11; WT-YFV samples *n* = 9; 17DD-YFV samples *n* = 13). To coat the plates, 8 µg/mL of the peptide diluted in coating buffer were added to Nunc MaxiSorp 96-well ELISA plates and incubated at 4 °C overnight. Next, the wells were washed three times using a washing buffer and a blocking buffer (2.5% *w*/*v* BSA, 2.5% *w*/*v* nonfat dry milk, 1X phosphate-buffered saline with 0.05% Tween20) were added followed by incubation at 37 °C for 1 h. After washing as described serum from each group, diluted in Standard Buffer for ELISA, (1:100) was added, followed by an incubation at 37 °C for 2 h. Anti-human IgG peroxidase conjugate (Sigma-Aldrich Corporation, Saint Louis, MO, USA), diluted in standard buffer for ELISA (1:10000) was added. After 1 h of incubation at 37 °C, the wells were washed and TMB Substrate Solution (Thermo Fisher Scientific, Waltham, MA, USA) was added to each well following by the addition of the Stop Solution. Absorbance was read on SpectraMax (Molecular Devices, San José, CA, USA) using a 450 nm wavelength.

### 2.5. Statistical Analysis

All statistical analyses were performed using GraphPad Prism 8 (Version 8, GraphPad Software, San Diego, CA, USA). A two-Way ANOVA with Geisser–Greenhouse correction and Tukey's multi-comparison test was used to select peptides differentially recognized between sera collected from infected or vaccinated individuals after microarray procedures. ELISA analysis was assessed using Kruskal–Wallis with Dunn’s multiple comparisons test. Differences were considered significant when *p*-value < 0.05.

## 3. Results

In concordance with observations by Gómez et al. (2018) [28], a high average identity score, greater than 99.9%, was observed, with the sequences of these isolates being almost identical throughout their length (the minimum identity observed was 0.99 and the maximum identity 100%, mean of identity 99.98%, standard deviation = 0.0149), and remarkably, independently from the host from which they were isolated, highlighting how challenging the search for peptides capable of being differentially recognized by antibodies present in the serum of individuals infected by the different strains of YFV can be (Table 1).

**Figure 1:**
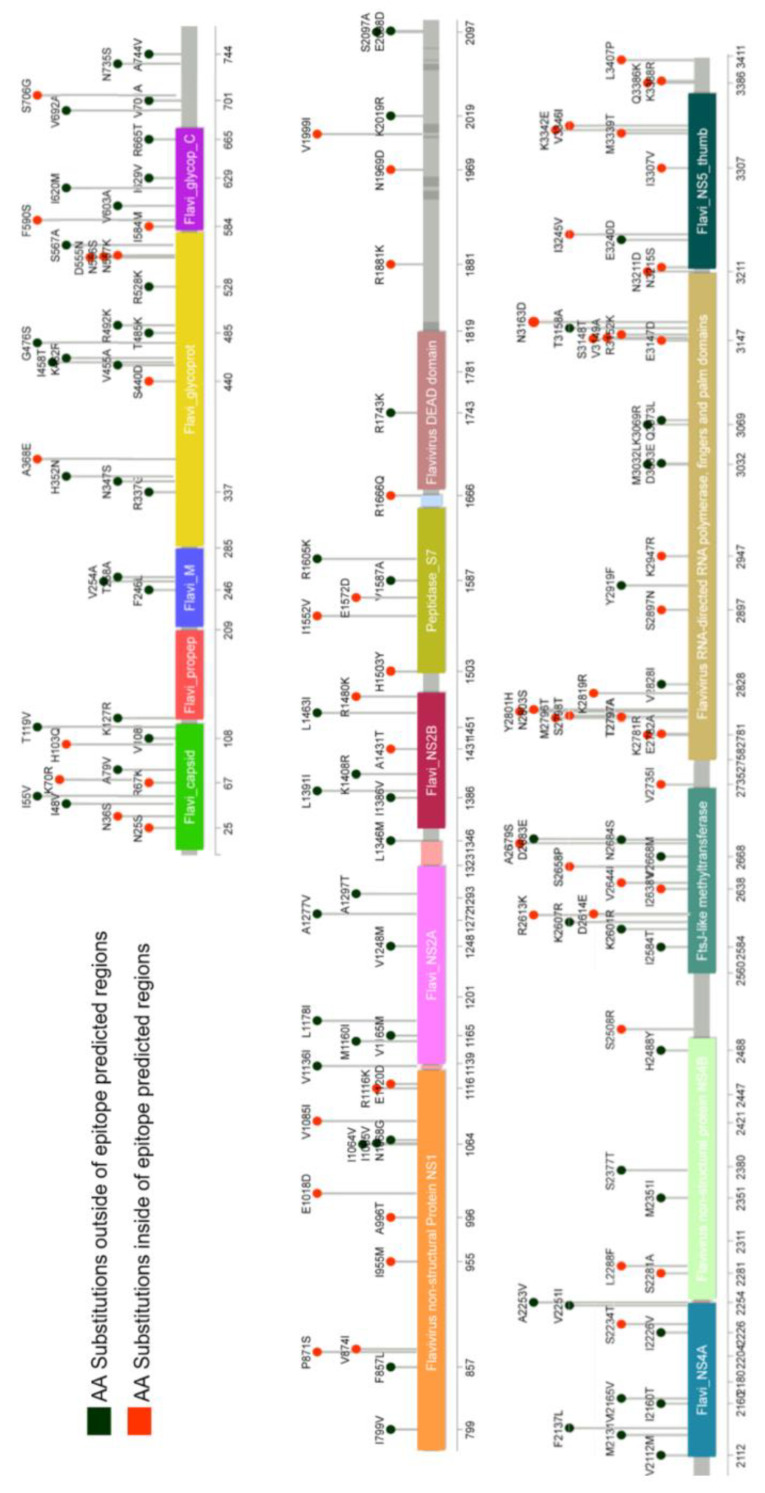
Distribution of amino acid substitutions in the YFV polyprotein. Using the protein sequence of the 17DD vaccine strain as a reference, differences in the amino acid sequences related to the 2017 isolate ES-505 were identified. The 144 substitutions identified are distributed throughout the virus polyprotein sequence.

Overall, an identity of approximately 95.7% was observed in WT-YFV and 17DD-YFV polyproteins. A total of 144 amino acid changes distributed throughout the polyprotein sequence were identified (Figure 1, Supplementary Table S1) with no apparent concentration of differences in specific regions or proteins. These identified differences were later used as a reference for peptide database construction.

A total of 222 peptides were designed (Supplementary Table S2), containing the alteration identified in the 2017 isolates and the corresponding peptide containing the amino acid variant of the 17DD strain.

**Figure 2:**
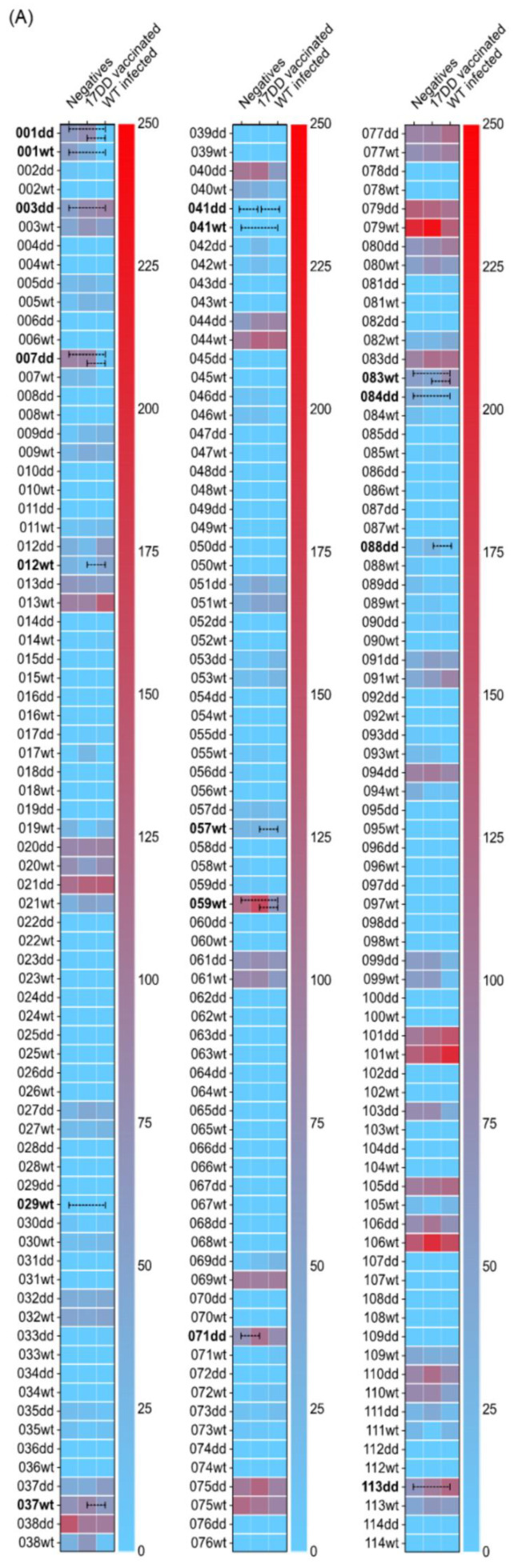

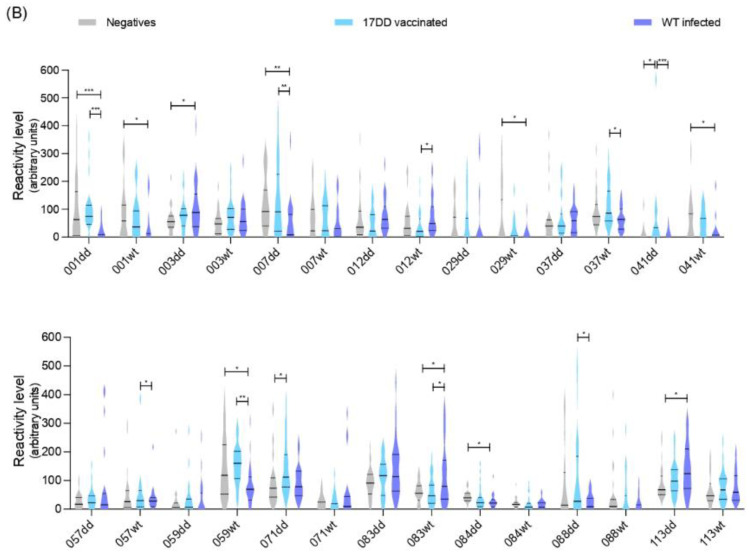
Recognition pattern of each tested peptide by antibodies in sera collected from YFV-vaccinated individuals, WT-infected individuals, or control sera (negatives). (**A**) Heatmap was generated by plotting the median obtained by the reading of each triplicate of the 222 peptides incubated with one of all the collected serum samples. Negative values were corrected to zero. A color scale representing all the reactivity levels was applied to represent the values ranging from zero (blue) to 250 (red). Statistical differences between groups were identified using two-Way ANOVA with Geisser–Greenhouse correction and Tukey multi-comparison (GraphPad Prism). Differences between groups with *p* < 0.05 are highlighted on the heatmap (in bold). Dashed bars represent statistical differences between groups. (**B**) Violin plot highlighting the statistical differences between groups identified using two-Way ANOVA with Geisser–Greenhouse correction and Tukey multi-comparison (GraphPad Prism). Graph was generated by plotting the median obtained from the reading of each triplicate of the 16 peptides incubated with one of all the collected serum samples. Negative values were corrected to zero. * = *p* < 0.05, ** = *p* < 0.01, *** = *p* < 0.001.

Most peptides were not differentially recognized by sera obtained from individuals naturally infected by the YFV or sera collected from YFV-vaccinated individuals. Among the 222 tested peptides, sixteen peptides showed to be differentially recognized (*p*-value < 0.05) at least between two of the three tested groups (Figure 2A). The peptides 012wt and 083wt, derived from the WT-YFV strain, showed higher signals with sera obtained from infected individuals. Similarly, the peptides 041dd and 088dd, derived from the 17DD-YFV strain, were more strongly recognized by sera collected from vaccinated individuals in comparison to the sera collected from infected individuals. Yet, antigen 083wt also exhibited a higher signal from sera of infected individuals when compared to sera from negative and from YFV vaccinated individuals than from infected individuals. On the other hand, peptides 037wt and 057wt, derived from WT-YFV strain, were better recognized by sera collected from vaccinated individuals. Moreover, peptides 041dd and 071dd were significantly better recognized by sera collected from vaccinated individuals than sera collected from negative individuals. Peptides 001dd, 001wt, 007dd, 029wt, 041wt, 059wt, and 084dd were significantly better recognized by sera obtained from negative individuals in contrast to sera collected from infected individuals. Regardless, 001dd, 007dd, and 059wt also showed a higher signal when incubated with sera from YFV vaccinated than infected individuals. Peptides 003dd and 113dd exhibited a more elevated signal with sera from infected individuals when compared to sera collected from negative individuals (Figure 2B).

**Figure 3:**
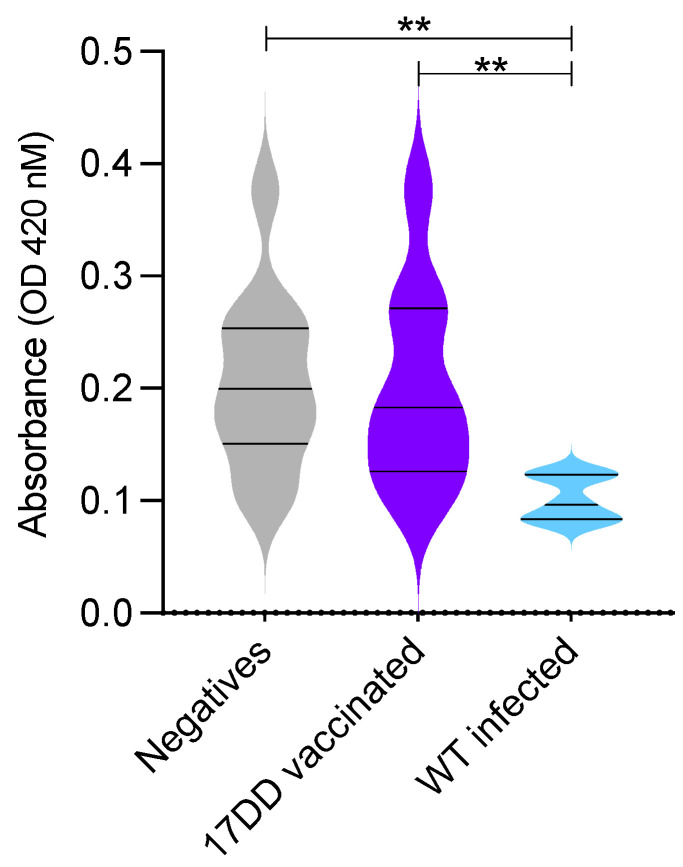
Recognition pattern of peptide 007dd from sera collected from different groups of individuals. ELISA was employed to verify if sera from 17DD-YFV individuals were more strongly recognized by 007dd peptide than WT-YFV infected individuals. Statistical differences among groups were assessed using Kruskal–Wallis with Dunn’s multiple comparisons test (GraphPad Prism). Differences between groups with *p* < 0.01 are represented with two asterisks (**) connected by dashed bars.

To validate the microarray results, the peptide 007dd was randomly selected (among the peptides derived from the 17DD-YFV strain that were better recognized by sera of vaccinated individuals when compared to infected individuals: 001dd, 007dd, 041dd, and 088dd) to be further explored by ELISA assay. As shown in Figure 3, a higher absorbance was observed for sera collected from negative or vaccinated individuals in comparison to sera from naturally infected individuals. 

## 4. Discussion

Since the early 1940’s, YFV has circulated, in Brazil, almost exclusively through a sylvatic cycle with mosquitos from the genus *Haemagogus* as the primary vector [35,36]. However, from 2016 to 2018, YFV outbreaks started appearing in the southeast region of Brazil, with a high mortality rate, which included urbanized areas, raising serious concerns about the increasing urbanization of sylvatic areas, the introduction of the transmission cycle to urban areas, and the possibility of involvement of mosquitos from the genus *Aedes*. One issue that became evident during the 2016–2018 outbreaks is the difficulty in the serological differentiation between vaccinated and infected individuals which is a major roadblock for the swift identification and control of outbreaks to mitigate its public health impact. 

In this study, we sought to find a way to serologically differentiate individuals that were vaccinated against Yellow Fever from those who were naturally infected. The main reason to establish this goal is based on the information gap concerning vaccination against YFV, which is based on self-informed vaccination and/or the vaccination certificate presented by the individuals, which, in many cases is lost, or contains incomplete information. In this context, it is reasonable to expect that for a large portion of the population, the immunization data is unreliable, a scenario that is even further complicated since the YFV vaccine is preconized as a single dose (or from ten-to-ten years) and first vaccine shots are usually givens among infants of six-month to two-year-old children and their records are often lost.

In the effort to identify epitopes useful to differentiate vaccinated from infected individuals, we have implemented a combination of in silico analysis of the YFV predicted protein to identify not only sequence differences between the vaccine strain (17DD-YFV) and the 2016–2018 circulating strain but also potential b-cell linear epitopes, with serological analysis using an advanced high throughput peptide array and ELISA assay. 

One of the main challenges in this type of approach is to identify useful amino acid variations between highly conserved sequences, and here we designed 222 peptides with this potential. Not surprisingly, most peptides were found to not be differentially recognized by vaccinated and infected individuals. With the elevated degree of sequence identity among the genome from the three strains, most of the designed peptides contained only one or two amino acid substitutions. However, a few peptides have shown promising results and should be further investigated.

Besides the YFV structural proteins, the NS1 protein is also able to trigger the host immune system, due to its secretion during the viral replication [37]. Thus, is expected that this work should consider only the YFV structural proteins and the NS1 protein. However, a considerable amount of amino acid differences between the 17DD vaccine strain and the wild-type circulating strains of YFV were identified in the non-structural proteins. Moreover, during the viral replication process, some infected host cells may lose their integrity and therefore expose atypical antigens as a consequence of the incomplete virion assembly. Additionally, the protein microarray is a high-throughput screening approach that allows the study of numerous peptides to be screened despite their regular access to the human immune system. Our analysis showed that, from more than 70 peptides designed based on the non-structural proteins, ten peptides were differentially recognized, at least, between two of the three groups: three were in NS1, one in NS2B, two in NS3, one in NS4A, and three in NS5.

Most notably, derived from the WT-YFV strain, the peptides 012wt and 083wt showed higher reactivity for infected individual sera while the antigens 001dd, 007dd, 041dd, and 088dd were found to react more strongly with sera derived from vaccinated individuals. This differential profile of antibody recognition is a good indication that it is feasible to separate infected individuals from vaccinated individuals serologically. To increase sensitivity and specificity, it is likely that a panel of antigens, as opposed to single peptides, would be necessary in a serological diagnosis context. Assis et al. 2021 demonstrated, by logistic regression analysis, an increase in sensitivity and specificity of a multi-antigen microarray to detect SARS-CoV-2 antibodies by combining antigens [38]. Thus, for efficient serological classification of Yellow Fever vaccinees or infected individuals, a broader study would be necessary for the determination of the optimal combination of antigens. 

In addition to the aforementioned peptides, a differential recognition between infected and vaccinees was also detected for the peptides 037wt, 057wt, and 059wt, derived from the WT-YFV strain. However, these antigens showed higher signals against sera from vaccinated individuals. This was not anticipated as these antigens are derived from WT-YFV. The reason for this observation is unknown, although the difference in reactivity is relatively small for the antigens 037wt and 057wt. Although not previously expected, the antigen 059wt can potentially be part of an antigen panel for differential diagnostics or may be useful as a vaccination marker. 

In this work, six peptides designed based on the WT-YFV polyprotein were significantly recognized by sera from YFV vaccinees and/or negative individuals. As expected, peptides designed based on the 17DD-YFV polyprotein were significantly recognized by sera from YFV vaccinees. However, high reactivity levels were observed when using sera collected from negative individuals. Brazil encounters a scenario characterized by the cocirculation of other Flavivirus as *Dengue virus* (DENV) and *Zika virus* (ZIKV) and the serum samples employed in the present study were only tested for YFV antibodies. Regarding that, some serum samples could exhibit antibodies against other flaviviruses [39,40]. Since YFV shares considerable identities in its polyprotein sequence with other flaviviruses, and consequently would share similar epitopes, a cross-rection among YFV antibodies against other flaviviruses can occur [41,42]. Moreover, the “Negative group” should be composed of volunteers with no record of vaccination or recent infection (see Section 2.1). However, vaccination records or patient information would be failing to properly characterize some individuals as non-vaccinated (e.g., patients with no vaccination certificate and with no memory of vaccination). Even so, YFV-17DD peptides recognized by vaccinated or negative individuals, such as the 007dd, still exhibited lower signal when exposed to sera of WT-YFV infected individuals, meaning that a panel of peptides behaving similarly to peptide 007dd would be used to differentiate vaccinated from naturally infected individuals.

Finally, the peptides 003dd, 083dd, and 113dd, derived from the vaccine strain, were observed to be more reactive against sera from infected individuals as opposed to the negative samples. These peptides were not found to be potentially differential between vaccinees and infected. However, these antigens have the potential to be used as positive serological markers for infection or to access the vaccine antigen titers. 

In summary, the data presented in this work is a preliminary exploration of potential peptides for the serological classification of vaccinees and infected individuals as well as a proof-of-concept for the application of the peptide microarray technology for a large-scale assessment of the serology of Yellow Fever. Here, we identified multiple antigens with the potential to be used in diagnostic serological tests, potentially as a comprehensive antigen panel. This is an important step toward the development of a fast and reliable test for the quick identification of exposed individuals during Yellow Fever outbreaks where the differentiation between infected and vaccinated individuals is vital for the proper control and public health management.

## Figures and Tables

**Table 1:** Sequence identity matrix of isolates from the 2016–2017 Yellow Fever outbreak.

	**AVQ67911.1**	**AVQ67912.1**	**AVQ67913.1**	**AVQ67914.1**	**AVQ67915.1**	**AVQ67916.1**	**AVQ94372.1**	**ES-505**	**AVQ94369.1**	**AVQ94370.1**	**AVQ94371.1**
**AVQ67911.1**	1										
**AVQ67912.1**	0.9997	1									
**AVQ67913.1**	0.9997	1	1								
**AVQ67914.1**	0.9997	1	1	1							
**AVQ67915.1**	1	0.9997	0.9997	0.9997	1						
**AVQ67916.1**	1	0.9997	0.9997	0.9997	1	1					
**AVQ94372.1**	1	0.9997	0.9997	0.9997	1	1	1				
**ES-505**	0.9997	1	1	1	0.9997	0.9997	0.9997	1			
**AVQ94369.1**	0.9997	1	1	1	0.9997	0.9997	0.9997	1	1		
**AVQ94370.1**	1	0.9997	0.9997	0.9997	1	1	1	0.99	0.9997	1	
**AVQ94371.1**	0.9997	1	1	1	0.9997	0.9997	0.9997	1	1	0.9997	1

## Data Availability

Not applicable.

## Supplementary Materials

The following supporting information can be downloaded at: https://www.mdpi.com/article/10.3390/v14081645/s1, Supplementary Table S1: Amino acid substitutions found when comparing WT-YFV (KY885001.2) and 17DD-YFV (U17066.1) polyproteins; Supplementary Table S2: YFV peptides designed and imprinted onto microarray slides and their respective information regarding amino acid position on the polyprotein, virus-encoded protein, and B-epitope prediction.
