# Supplementary material for: Mapping and Validation of Peptides Differentially Recognized by Antibodies from the Serum of Yellow Fever Virus-Infected or 17DD-Vaccinated Patients
Source: Viruses. 2022 Jul 27;14(8):1645. doi: 10.3390/v14081645 (PMC9415205; doi:10.3390/v14081645)
Supplement: Supplementary file 1 [file viruses-14-01645-s001.zip › Supplementary Table S1.pdf]

Supplementary Table S1. Amino acid substitutions found when comparing WT-YFV (KY885001.2) and 17DD-YFV (U17066.1) polyproteins

Protein	Substitutions
Capsid	N25S, N36S, I48V, I55V, R67K, K70R, A79V, H103Q, V108I, T119V
prM/M	K127R, F246L, V254A, T258A
Env	R337G, N347S, H352N, A368E, S440D, V455A, I458T, K462R, G476S, T485K, R492K, R528K, D555N, N556S, N557K, S567A, I584M. F590S, V603A, I620M, I629V, R665T, V692A, V701A, S706G, N735S, A744V
NS1	I799V, F857L, P871S, V874I, I955M, A996T, E1018D, I1064V, I1065V, N1068G, V1085I, R1116K, E1120D
NS2A	V1136I, M1160I, V1165M, L1178I, V1248M, T1277V, A1297T, A1302T, F1313S, L1346M
NS2B	I1386V, L1391I, K1408R, A1431T, L1463I, R1480K
NS3	H1503Y, I1552V, E1572D, V1587A, R1605K, R1666Q, R1743K, R1881K, N1969D, V1999I, K2019R, S2097A, E2098D
NS4A	V2112M, M2131V, F2137L, I2160T, I2165V, I2226V, S2234T, V2251I, A2253V
NS4B	S2281A, L2288F, M2351I, S2377T, H2488Y
NS5	S2508R, I2584T, K2601R, K2607R, R2613K, D2614E, I2638V, V2644I, S2658P, V2668M, A2679S, D2683E, N2684S, V2735I, K2781R, E2782A, M2796T, T2797A, S2798T, Y2801H, N2803S, K2819R, V2828I, S2897N, Y2919F, K2947R, M3032L, D3033E, K3069R, Q3073L, E3147D, S3148T, V3149A, R3152K, T3158A, N3163D, N3211D, N3215S, E3240D, I3245V, I3307V, M3339T, K3342E, V3346I, Q3386K, K3388R, L3407P
