# Supplementary material for: Mapping and Validation of Peptides Differentially Recognized by Antibodies from the Serum of Yellow Fever Virus-Infected or 17DD-Vaccinated Patients
Source: Viruses. 2022 Jul 27;14(8):1645. doi: 10.3390/v14081645 (PMC9415205; doi:10.3390/v14081645)
Supplement: Supplementary file 1 [file viruses-14-01645-s001.zip › Supplementary Table S2.pdf]

Supplementary Table S2. YFV peptides designed and imprinted onto microarray slides and their respective information regarding amino acid position on the polyprotein, virus-encoded protein, and B-epitope prediction.

YFV-17dd Peptides						YFV-WT Peptides					
Peptide name	Position	Peptide sequence	B-epitope prediction (Y/N)	B-epitope score	YFV protein	Peptide name	Position	Peptide sequence	B-epitope prediction (Y/N)	B-epitope score	YFV protein
001dd	19-30	GVRSLSSKIKQK	Y	0,65	Capsid	001wt	19-30	GVRSLSNKIKQK	Y	0,66	Capsid
002dd	31-42	TKQIGSRPGPSR	Y	0,63	Capsid	002wt	31-42	TKQIGNRPGPSR	Y	0,63	Capsid
003dd	43-54	GVQGFVFFFLFN	N	0,34	Capsid	003wt	43-54	GVQGFIFFFLFN	N	0,32	Capsid
004dd	50-61	FFLFNVLTGKKI	N	0,41	Capsid	004wt	50-61	FFLFNILTGKKI	N	0,39	Capsid
005dd	46-57	GFVFFFLFNVLT	N	0,34	Capsid	005wt	46-57	GFIFFFLFNILT	N	0,32	Capsid
006dd	63-74	AHLKKLWRMLDP	Y	0,53	Capsid	006wt	63-74	AHLKRLWKMLDP	Y	0,54	Capsid
007dd	74-85	PRQGLVVLRKVK	N	0,45	Capsid	007wt	74-85	PRQGLAVLRKVK	N	0,46	Capsid
008dd	67-79	KLWRMLDPRQGLV	Y	0,52	Capsid	008wt	67-79	RLWKMLDPRQGLA	Y	0,53	Capsid
009dd	100-111	RRSQDVLTIQFL	Y	0,53	Capsid	009wt	100-111	RRSHDVLTVQFL	Y	0,53	Capsid
010dd	110-121	FLILGMLLMVGG	N	0,28	Capsid	010wt	110-121	FLILGMLLMTGG	N	0,30	Capsid
011dd	122-133	VTLVRRNRWLLL	N	0,40	preM	011wt	122-133	VTLVRKNRWLLL	N	0,41	preM
012dd	240-251	QKIERWLVRNPF	N	0,43	preM	012wt	240-251	QKIERWFVRNPF	N	0,41	preM
013dd	250-261	PFFAATALAAY	N	0,26	preM	013wt	250-261	PFFAVTALTIAY	N	0,26	preM
014dd	246-258	LVRNPFFAATALA	N	0,30	preM	014wt	246-258	FVRNPFFAVTALT	N	0,30	preM
015dd	332-343	TVAIDGPAEARK	N	0,46	Env	015wt	332-343	TVAIDRPAEARK	N	0,47	Env
016dd	344-355	VCYSAVLTNVKI	N	0,45	Env	016wt	344-355	VCYNAVLTHVKI	N	0,46	Env
017dd	363-374	GEAHLEEENEGD	Y	0,60	Env	017wt	363-374	GEAHLAEENEGD	Y	0,60	Env
018dd	435-446	ENWNTDIKTLKF	Y	0,55	Env	018wt	435-446	ENWNTSIKTLKF	Y	0,56	Env
019dd	453-464	QEAEFTGYGRAT	N	0,46	Env	019wt	453-464	QEVEFIGYGKAT	N	0,46	Env
020dd	471-482	TAVDFSNSYIAE	N	0,46	Env	020wt	471-482	TAVDFGNSYIAE	N	0,46	Env
021dd	483-494	MEKESWIVDKQW	N	0,46	Env	021wt	483-494	METESWIVDRQW	N	0,44	Env
022dd	522-533	PHAATIKVLALG	N	0,46	Env	022wt	522-533	PHAATIRVLALG	N	0,46	Env
023dd	550-561	TKDTNNSKLYKL	Y	0,53	Env	023wt	550-561	TKDTNDNNLYKL	Y	0,55	Env
024dd	562-573	HGGHVACRVKLS	N	0,41	Env	024wt	562-573	HGGHVSCRVKLS	N	0,42	Env
025dd	582-593	YKMCTDKMSFVK	Y	0,53	Env	025wt	582-593	YKICTDKMFFVK	Y	0,51	Env
026dd	597-608	DTGHGTAVMQVK	N	0,46	Env	026wt	597-608	DTGHGTVVMQVK	N	0,46	Env
027dd	615-626	CRIPVMVADDLT	N	0,44	Env	027wt	615-626	CRIPVIVADDLT	N	0,44	Env
028dd	624-635	DLTAAVNKGILV	N	0,43	Env	028wt	624-635	DLTAAINKGILV	N	0,43	Env
029dd	619-630	VMVADDLTAAVN	N	0,48	Env	029wt	619-630	VIVADDLTAAIN	N	0,47	Env
030dd	659-670	SYIIVGTGDSRL	N	0,39	Env	030wt	659-670	SYIIVGRGDSRL	N	0,40	Env
031dd	686-697	TQTMKGAERLAV	N	0,42	Env	031wt	686-697	TQTMKGVERLAV	N	0,42	Env
032dd	698-709	MGDAAWDFGSAG	Y	0,50	Env	032wt	698-709	MGDVAWDFSSAG	N	0,49	Env
033dd	691-702	GAERLAVMGDAA	N	0,42	Env	033wt	691-702	GVERLAVMGDVA	N	0,42	Env
034dd	729-740	GLFGGLSWITKV	N	0,46	Env	034wt	729-740	GLFGGLNWITKV	N	0,46	Env
035dd	739-750	KVIMGVVLIWVG	N	0,28	Env	035wt	739-750	KVIMGAVLIWVG	N	0,27	Env
036dd	734-745	LSWITKVIMGVV	N	0,37	Env	036wt	734-745	LNWITKVIMGAV	N	0,36	Env
037dd	794-805	GDGIFVFRDSD	N	0,45	NS1	037wt	794-805	GDGIFIFRDSDD	N	0,45	NS1
038dd	852-863	EINAILEENEVD	N	0,43	NS1	038wt	852-863	EINAIFEENEVD	N	0,44	NS1
039dd	867-878	VVQDSKNIYQRG	Y	0,55	NS1	039wt	867-878	VVQDPKNVYQRG	Y	0,57	NS1
040dd	950-961	VFEYTMDCDGSI	N	0,48	NS1	040wt	950-961	VFEYTIDCDGSI	N	0,47	NS1
041dd	991-1002	IHTLETLDYKEC	N	0,49	NS1	041wt	991-1002	IHTLEALDYKEC	Y	0,50	NS1
042dd	1012-1023	TSVEESDMFMPR	Y	0,54	NS1	042wt	1012-1023	TSVEESEMFMPR	Y	0,54	NS1
043dd	1061-1072	TSVVVDGGCDGR	N	0,48	NS1	043wt	1061-1072	TSVIIDGNCDGR	N	0,49	NS1
044dd	1079-1090	TTDSGKIIPEWC	Y	0,54	NS1	044wt	1079-1090	TTDSGKVIPEWC	Y	0,55	NS1
045dd	1112-1123	EIRPKKTHDSHL	N	0,49	NS1	045wt	1112-1123	EIRPRKTHESHL	Y	0,50	NS1
046dd	1131-1142	GEIHAIPFGLVS	N	0,38	NS2a	046wt	1131-1142	GEIHAVPFGLVS	N	0,39	NS2a
047dd	1157-1158	PKQILVGGMVLL	N	0,34	NS2a	047wt	1157-1158	PKQMLVGGVVLL	N	0,35	NS2a
048dd	1173-1184	VGQVTILDLLKL	N	0,36	NS2a	048wt	1173-1184	VGQVTLLDLLKL	N	0,38	NS2a
049dd	1243-1254	IALGGMMGGLWK	N	0,39	NS2a	049wt	1243-1254	IALGGVMGGLWK	N	0,42	NS2a
050dd	1272-1283	RKASNVILPLMA	N	0,33	NS2a	050wt	1272-1283	RKASNTILPLMA	N	0,33	NS2a
051dd	1294-1305	RLATMLFCTVVI	N	0,29	NS2a	051wt	1294-1305	RLAAMLFCAVVI	N	0,29	NS2a
052dd	1308-1319	VLHQNSKDTSMQ	N	0,45	NS2a	052wt	1308-1319	VLHQNFKDTSMQ	N	0,45	NS2a
053dd	1341-1352	GLCAFMATRIFG	N	0,37	NS2a	053wt	1341-1352	GLCAFLATRIFG	N	0,37	NS2a
054dd	1383-1394	LGPVAVGGILMM	N	0,27	NS2b	054wt	1383-1394	LGPIAVGGLLMM	N	0,28	NS2b
055dd	1402-1413	VDGLELRKLGEV	N	0,49	NS2b	055wt	1402-1413	VDGLELKKLGEV	N	0,49	NS2b
056dd	1425-1436	SARYDVTLSEQG	Y	0,52	NS2b	056wt	1425-1436	SARYDVALSEQG	Y	0,54	NS2b
057dd	1458-1469	LVGAAIHPFALL	N	0,27	NS2b	057wt	1458-1469	LVGAALHPFALL	N	0,28	NS2b
058dd	1473-1484	AGWLFHVKGARR	N	0,42	NS2b	058wt	1473-1484	AGWLFHVRGARR	N	0,43	NS2b
059dd	1494-1505	TPKIIEECEYLE	Y	0,57	NS3	059wt	1494-1505	TPKIIEECEHLE	Y	0,58	NS3
060dd	1546-1557	RNGKKLVPSWAS	Y	0,51	NS3	060wt	1546-1557	RNGKKLIPSWAS	Y	0,54	NS3
061dd	1567-1578	GSWKLDGRWDGE	Y	0,56	NS3	061wt	1567-1578	GSWKLEGRWDGE	Y	0,56	NS3
062dd	1582-1593	QLIAAAPGKNVV	N	0,47	NS3	062wt	1582-1593	QLIAAVPGKNVV	N	0,44	NS3
063dd	1599-1610	PSLFKVKNGGEI	N	0,47	NS3	063wt	1599-1610	PSLFKVRNGGEI	Y	0,55	NS3
064dd	1660-1671	EGKEELQEIPTM	Y	0,53	NS3	064wt	1660-1671	EGKEELREIPTM	Y	0,53	NS3
065dd	1738-1749	AHGSGKEVIDAM	N	0,45	NS3	065wt	1738-1749	AHGSGREVIDAM	N	0,44	NS3
066dd	1877-1888	KTFEKEYPTIKQ	Y	0,54	NS3	066wt	1877-1888	KTFEREYPTIKQ	Y	0,54	NS3
067dd	1964-1975	EPTSEDNAHHVC	Y	0,61	NS3	067wt	1964-1975	EPTSENNAHHVC	Y	0,60	NS3
068dd	1994-2005	APLYGIEGTKTP	Y	0,52	NS3	068wt	1994-2005	APLYGVEGTKTP	Y	0,52	NS3
069dd	2013-2024	LRDDQRRVFREL	N	0,49	NS3	069wt	2013-2024	LRDDQRKVFREL	N	0,48	NS3
070dd	2092-2103	DQSALADFIKFA	Y	0,50	NS3	070wt	2092-2103	DQSALSEFIKFA	Y	0,51	NS3
071dd	2108-2119	GAAEMLVVLSEL	N	0,41	NS4a	071wt	2108-2119	GAAEVLVVLSEL	N	0,40	NS4a
072dd	2129-2140	EAVDTISVLLHS	N	0,35	NS4a	072wt	2129-2140	EAMDTISVFLHS	N	0,38	NS4a
073dd	2157-2168	AMTTVMLFVLAG	N	0,24	NS4a	073wt	2157-2168	AMTIVMLFILAG	N	0,23	NS4a
074dd	2220-2231	VLMVVVVPEPGQ	N	0,34	NS4a	074wt	2220-2231	VLMVVVIPEPGQ	N	0,35	NS4a
075dd	2234-2245	TIQDNQVAYLII	N	0,40	2kP	075wt	2234-2245	SIQDNQVAYLII	N	0,40	2kP
076dd	2245-2256	IGILTLISVVAA	N	0,18	2kP	076wt	2245-2256	IGILTLVSAVAA	N	0,19	2kP
077dd	2275-2286	LIPSSAAPWSWP	Y	0,58	NS4b	077wt	2275-2286	LIPSSASPWSWP	Y	0,60	NS4b
078dd	2283-2294	WSWPDFDLKPGA	Y	0,51	NS4b	078wt	2283-2294	WSWPDLDLKPGA	Y	0,52	NS4b
079dd	2279-2290	SAAPWSWPDFDL	Y	0,56	NS4b	079wt	2279-2290	SASPWSWPDLDL	Y	0,57	NS4b
080dd	2346-2357	NISVIILLVSGW	N	0,30	NS4b	080wt	2346-2357	NISVIMLLVSGW	N	0,30	NS4b
081dd	2371-2382	CAMLHWTLILPG	N	0,29	NS4b	081wt	2371-2382	CAMLHWSLILPG	N	0,29	NS4b
082dd	2483-2494	VMRGNYYAFVGV	N	0,31	NS4b	082wt	2483-2494	VMRGNHYAFVGV	N	0,32	NS4b
083dd	2507-2518	GRANGKTLGEVW	Y	0,57	NS5	083wt	2507-2518	GSANGKTLGEVW	Y	0,58	NS5
084dd	2579-2590	LEGRVTDLGCGR	N	0,41	NS5	084wt	2579-2590	LEGRVIDLGCGR	N	0,39	NS5
085dd	2596-2607	YAAAQREVSGVR	N	0,41	NS5	085wt	2596-2607	YAAAQKEVSGVK	N	0,48	NS5
086dd	2605-2616	GVRGFTLGKEGH	Y	0,53	NS5	086wt	2605-2616	GVKGFTLGRDGH	N	0,49	NS5
087dd	2635-2646	KTDVHRLEPIKC	Y	0,52	NS5	087wt	2635-2646	KTDIHRLEPVKC	Y	0,51	NS5
088dd	2655-2666	ESSPSSVTEGER	Y	0,58	NS5	088wt	2655-2666	ESSSSSVTEGER	Y	0,57	NS5
089dd	2662-2673	TEGERTMRVLDT	Y	0,50	NS5	089wt	2662-2673	TEGERTVRVLDT	N	0,47	NS5
090dd	2657-2668	SPSSVTEGERTM	Y	0,57	NS5	090wt	2657-2668	SSSSVTEGERTV	Y	0,56	NS5
091dd	2676-2687	KWLSCGVESFCV	N	0,44	NS5	091wt	2676-2687	KWLACGVDNFCV	N	0,44	NS5
092dd	2730-2741	GARSNITFTVNQ	Y	0,51	NS5	092wt	2730-2741	GARSNVTFTVNQ	Y	0,51	NS5
093dd	2776-2787	KGPLDRAAIEER	Y	0,56	NS5	093wt	2776-2787	KGPLDKEAIEER	Y	0,55	NS5
094dd	2794-2805	EYTATWFHDSDN	Y	0,54	NS5	094wt	2794-2805	EYMTSWFYDNDN	Y	0,55	NS5
095dd	2813-2824	CGSYVTRTSGSA	Y	0,50	NS5	095wt	2813-2824	CGSYVTKTSGSA	Y	0,51	NS5
096dd	2823-2834	SAASMINGVIKI	N	0,43	NS5	096wt	2823-2834	SAASMVNGVIKI	N	0,43	NS5
097dd	2818-2829	TRTSGSAASMIN	Y	0,50	NS5	097wt	2818-2829	TKTSGSAASMVN	Y	0,50	NS5
098dd	2892-2903	LAREKNPRLCTK	Y	0,51	NS5	098wt	2892-2903	LAREKSPRLCTK	Y	0,51	NS5
099dd	2914-2925	AAIGAFLEEQEQ	Y	0,52	NS5	099wt	2914-2925	AAIGAYLEEQEQ	Y	0,52	NS5
100dd	2942-2953	VDEERRLHQQGR	Y	0,55	NS5	100wt	2942-2953	VDEERKLHQQGR	Y	0,55	NS5
101dd	3027-3038	RDLAALEGGGFY	N	0,45	NS5	101wt	3027-3038	RDLAAMDGGGFY	N	0,45	NS5
102dd	3065-3076	SPHHRKLALAVM	N	0,42	NS5	102wt	3065-3076	SPHHKKLAQAVM	N	0,43	NS5
103dd	3144-3155	DCDDTALTKLEA	Y	0,53	NS5	103wt	3144-3155	DCDESVLTRLEA	Y	0,51	NS5
104dd	3155-3166	AWLAEHGCDRLK	N	0,48	NS5	104wt	3155-3166	AWLTEHGCNRLK	N	0,48	NS5
105dd	3147-3158	DTALTKLEAWLA	Y	0,50	NS5	105wt	3147-3158	ESVLTRLEAWLT	N	0,49	NS5
106dd	3207-3218	SKGWDDWESVPF	Y	0,55	NS5	106wt	3207-3218	SKGWNDWENVPF	Y	0,55	NS5
107dd	3237-3248	PCRDQDELVGRG	N	0,49	NS5	107wt	3237-3248	PCREQDELIGRG	N	0,45	NS5
108dd	3301-3312	GRTTWSVHGKGE	Y	0,56	NS5	108wt	3301-3312	GRTTWSIHGKGE	Y	0,57	NS5
109dd	3337-3348	KTTVKEWRDIPY	Y	0,56	NS5	109wt	3337-3348	KTMVKKWRDVPY	Y	0,58	NS5
110dd	3382-3393	TLIGKERYTDYL	Y	0,51	NS5	110wt	3382-3393	TLIGQEKYTDYL	Y	0,51	NS5
111dd	3399-3410	YSVDADLQPGEL	Y	0,56	NS5	111wt	3399-3410	YSVDADLQLGEL	Y	0,57	NS5
112dd	23-38	LSSKIKQKTKQIGSR	Y	0,67	Capsid	112wt	23-38	LSNKIKQKTKQIGNR	Y	0,67	Capsid
113dd	1605-1619	KNGGEIGAVALDYPS	Y	0,50	NS3	113wt	1605-1619	RNGGEIGAVALDYPS	Y	0,58	NS3
114dd	2624-2638	SLGWNIITFKDKTDV	Y	0,50	NS5	114wt	2624-2638	SLGWNIITFKDKTDI	Y	0,55	NS5

Amino acids in red indicate the amino acid differences between the YFV-17DD and YFV-WT strains
